# Embracing Uncertainty to Enable Transformation: The Process of Engaging in Trialogue for Mental Health Communities in Ireland

**DOI:** 10.5334/ijic.3085

**Published:** 2018-04-18

**Authors:** Simon Dunne, Liam MacGabhann, Paddy McGowan, Michaela Amering

**Affiliations:** 1 School of Nursing and Human Sciences, Dublin City University, Dublin, IE; 2 Expert by Experience, Mental Health Consultant, IE; 3 Department of Psychiatry and Psychotherapy, Medical University of Vienna, Vienna, AT

**Keywords:** Open Dialogue, Trialogue, community-based participatory approach, mental health

## Abstract

**Introduction:**

Community-based participatory approaches are valuable methods for improving outcomes and effectively integrating care among mental health communities. Trialogue is one such approach which uses Open Dialogue methods with groups of three or more people from different backgrounds who deal with mental health systems.

**Theory and Method:**

The current study employed a participatory action research design, which prospectively documented the processes and challenges of participating in Trialogue Meetings. Individuals from participating communities took part in interviews, focus groups or Open Dialogue discussions across three cycles of research.

**Results:**

Three prospective themes were identified from participants’ dialogue across the three cycles of research relating to the experience of participating in Trialogue, the development of Open Dialogue skills and the growth of individual Trialogue communities.

**Conclusions and Discussion:**

The findings demonstrate that, where desirable conditions are present, Trialogue Meetings are worthwhile and sustainable community-based participatory approaches which encourage disclosure and dialogue surrounding mental health, and may assist in improved integration of care between mental health stakeholders. In particular, Trialogue Meetings stimulate the development of Open Dialogue skills, provide a platform for “vital” and “transformative” self-expression with the potential for positive mental health outcomes and may facilitate the growth of communities surrounding mental health.

## Introduction

Community-based participatory approaches are increasingly being recognised as valuable methods for improving mental health service outcomes [1,2,3]. Such approaches are based on the notion that communities are social groups with a collective identity, rather than territorial groups requiring physical proximity [4,5]. Participatory approaches involving such communities are typically designed to promote knowledge exchange within and between these groups and academic, policy-making or service delivery institutions [6] and have the potential to provide powerful learning experiences for participants, enable disempowered groups to address power imbalances and give voice to diverse perspectives surrounding mental health [1]. Furthermore, certain forms of these approaches are specifically designed to create participatory collaborative processes through which more integrated approaches to mental health care, and communities themselves, can develop [7,8].

One such burgeoning community-based participatory approach in mental health contexts is Open Dialogue [e.g. 9,10]. This approach is based on the work of Bakhtin [11], who conceived dialogue as a form of communication with the potential to bring about mutual understanding through the formation of a space, where people share experiences and co-construct meaning together. In an Open Dialogue, each participant is given the opportunity to participate in the conversation in their own way [10]. Such approaches have been successfully applied to mental health contexts, where they have been demonstrated to have the potential to act as therapeutic processes which underpin mental health services [9], strengthen relations between service users, service providers and academics [12] and enable equitable and effective organizational development through accommodation of a range of diverse perspectives [13].

Trialogue Meetings extend such approaches to groups of three or more people who deal with mental health systems; comprising service users, service providers and family members/friends [14]. Trialogue Meetings use Open Dialogue methods to allow individuals from each of these groups to participate in conversations surrounding mental health and their care and enable the creation of a common language and mutual understanding around such topics [15]. All participants involved in so-called “trialogic” communications attempt to create a shared reality that is mutually acceptable and accessible to them [15]. The first “Vienna Trialogue” was established in 1994 following the ground-breaking model of psychosis-seminars, which were developed in Germany in 1989 [16]. Since then, well over 150 Trialogue Meeting groups have been established in Germany, Austria, Switzerland, Poland, Turkey, Trinidad, China, US, Toronto UK and Ireland [14,15, 17,18]. In addition to anecdotal reports from participants that engaging in Trialogue Meetings are positive and transformative experiences [14,15,16, 18,19], two small-scale evaluations [20,21] and one large-scale national evaluation [22] have demonstrated that Trialogue participation can result in improved outcomes such as lower anxiety and enhanced communication skills. However, in spite of such promising findings, there is a dearth of research on the specific processes involved in participating in Trialogue Meetings in mental health contexts. In light of this, the current study was designed to describe the processes and challenges of participating in Trialogue Meetings for individuals from mental health communities in Ireland. A central objective of the study was to examine the potential for Trialogue Meetings to improve relations between service users, service providers and family members/friends; an outcome relating to the effective collaboration between formal mental health systems and local community settings in the delivery of care to service users.

## Methods

### Mental Health Trialogue Network Ireland

The Mental Health Trialogue Network Ireland was initiated as a community-based participatory project by a team of researchers in Dublin City University (DCU) in 2010 at the request of seven communities throughout Ireland. Local teams were established by one or two people in each community who had participated in a national mental health leadership service improvement programme and were assisted by the project team in DCU in establishing, moderating and recruiting for seven monthly Trialogue Meetings in each community over a one-year period. The resulting Trialogue Meetings consisted of service users, their family/friends, service providers and interested community members who agreed to meet on a monthly basis to discuss topics surrounding mental health issues in local community centres. Two workshop days were held in DCU, where further knowledge and skills were developed for Trialogue Meeting facilitators with a view to handing ownership of Trialogue over to the local communities. In order to encourage open discussion between participants from different perspectives, all Trialogue Meetings were conducted in a spirit of anonymity, where participants did not need to reveal their identity to other members of the group. Furthermore, the moderators of these meetings, who were local community members, implemented Open Dialogue ground rules; e.g. participants agreed that everyone had an equal voice in the conversation and that their diverse experiences carried equal weight.

### Design, Data Collection and Materials

This paper describes a Participatory Action Research [PAR] study (in line with a common definition of this method [8]), which prospectively documented the initial establishment of Trialogue Meetings in participating communities in Ireland. As a PAR project, all decisions surrounding the ideation, design and implementation of the project were carried out by participating community members in collaboration with the core research team. The qualitative data described herein was collected at three time-points (or cycles) across the study period. These cycles also represent three targeted points of structured ethical reflection [e.g. 23], where community members and research team members reflected on all aspects of Trialogue Meetings up to that time and re-negotiated processes of recruitment, data collection methods (including consent) and project outputs. All research participants were made aware of the process of consent in advance of each cycle of data collection and that data was being used with a view to producing research outputs relating to evaluating and documenting the process of Trialogue.

In Cycle 1, interview data was collected from 42 Trialogue participants after their first or second Trialogue Meeting. Interviews were structured around open-ended questions concerning participants’ experiences of key mental health issues such as knowledge surrounding mental health, existing mental health services, mental health stigma and responsibility for mental health and their experiences of participating in the initial Trialogue Meetings. Cycle 1 data was also collected at a first facilitator workshop training day in DCU, where 13 local community members from each constituency participated in a focus group with three members of the DCU team. This focus group centred on participants’ perspectives of the emerging story of Trialogue in each participating community up to that point. The Cycle 2 data represents focus group material which was collected at the second facilitator workshop training day after the fourth or fifth Trialogue Meeting had taken place in each community. In this focus group, ten community members from six remaining participating communities and two project team members discussed their experiences of Trialogue Meetings and the challenges which they had encountered up to that point. All data from Cycles 1 and 2 was audio-recorded and transcribed with participants’ consent.

Cycle 3 data was derived from the seventh and final set of Trialogue Meetings across the six remaining participating communities. These communities agreed to use these meetings as a basis for discussing the successes and failures of Trialogue and issues relating to its future sustainability. Participant consent took the form of process consent, whereby participants decided upon their level of participation and engagement in the final Trialogue Meeting. In keeping with the spirit of anonymity of the Trialogue Meetings, detailed hand-written notes were taken of these conversations by a designated transcriber in lieu of audio-recordings. This transcriber had been appropriately trained in qualitative research and field note-taking; she recorded all participants’ comments almost verbatim in short-hand notes and then fully transcribed the notes following the Trialogue Meetings. Once the notes were fully transcribed, the transcriber then met with the moderator for each Trialogue Meeting who cross-checked the notes to ensure their accuracy and quality.

### Data Analysis

The qualitative data from Cycle 1 was subjected to thematic analysis by the first author; an experienced qualitative researcher who had no previous contact with participants. This independent analysis was a measure taken to enhance the trustworthiness of the data analysis, as a common criticism of PAR is that findings may not be trustworthy given that the researchers were themselves participating in the study [e.g. 24]. The first author purposefully analysed data from this cycle for material relating to the processes and process outcomes involved in engaging in Trialogue Meetings. The three key themes which emerged from this analysis were subsequently used to purposefully identify qualitative themes relating to Trialogue processes from Cycle 2 and Cycle 3. This process was employed in order to track the progress of these three key processes of Trialogue across the study period. In addition to the above, the process of generating themes loosely followed the six-stage model of analysis outlined by Braun and Clarke [25]. At each stage of analysis, the credibility of this analysis process was enhanced by the second author (a member of the core research team), who cross-checked the quotes and themes to ensure they made sense and reflected Trialogue participants’ main concerns in relation to Trialogue processes.

## Results

At least 318 individuals participated in one or more Trialogue Meetings across the study period. Table 1 provides available demographic information for participants from Cycles 1–3.

**Table 1:** Demographic characteristics of participants at each cycle of the research process.

Cycle 1 Interviews		Cycle 1 Focus Group		Cycle 2 Focus Group		Cycle 3 Trialogue Meeting	

*Variable*	*N*	*Variable*	*N*	*Variable*	*N*	*Variable*	*N*

Total	42	Total	17	Total	13	Total	42
Gender		Gender		Gender		Gender	
Male	17	Male	5	Male	8	Male	24
Female	25	Female	11	Female	5	Female	18
Age		Unknown	1			Age	
16–24	2					16–24	2
25–35	10					25–35	10
35–45	8					35–45	8
45–55	18					45–55	18
55–65	3					55–65	3
65+	1					65+	1
Employment status						Employment status	
Currently employed	17					Currently employed	17
Unemployed	10					Unemployed	10
Student/in-training	6					Student/in-training	6
Self-employed	5					Self-employed	5
Home-maker	3					Home-maker	3
Retired/pensioner	1					Retired/pensioner	1
Mental health role						Mental health role	
Service user	11					Service user	12
Service provider	8					Service provider	8
Family/carer	1					Family/carer	3
Community member	3					Community member	3
Other	16					Other	16

The following analysis documents the evolution of three key process-relevant themes relating to Trialogue across the three cycles of the research process: the experience of participating in Trialogue, the development of Open Dialogue skills and the growth of individual Trialogue communities. These themes are presented below for each cycle together with excerpts from participant’s dialogue. Where quotations have been contracted, ellipses have been inserted in square brackets.

### Cycle 1

For Cycle 1 and 2 data, participants are identified by the data collection method and a corresponding number as follows: Interview Respondent 1 [IR.1], Focus Group 1 Respondent 1 [FGR1.1], and Focus Group 2 Respondent 1 [FGR2.1].

#### Entering a comfortable space: initial experiences of participating in Trialogue

Although there was some nervousness and tension initially, many people described their introductory experiences to Trialogue Meetings positively. Trialogue Meetings were seen as comfortable spaces, characterised by a welcoming attitude, where there was less of a chance of being “railroaded” in discussions.

“I felt very welcome and very accepted and I felt very positive attitude here to everyone and most people here had an input and they had a voice.” [IR.3]

This initial sense of comfort enabled participants to express themselves in a manner in which they felt they were typically unable to do (e.g. at home) and allowed them to discuss experiences that they may have often thought about but never discussed.

“There was stuff I was being brought back to think about; I said things that I probably haven’t even thought about for years. Like that thing that I said about [hospital], that’s something I’ve often put in my head, but it’s not something I talk about much.” [IR.12]

However, one member of the project team noted that, in spite of this process of “getting things off one’s chest” through dialogue, it was important not to regard Trialogue Meetings as a form of therapy.

“It can be very easy to mistake [Trialogue] for therapy and also you have therapist other group members that might think that they need to understand therapy mechanisms… But that is what it is exactly not, and that’s the richness of it, and maybe “shared ownership” is an important concept to keep us from this misunderstanding.” [FGR1.2]

Nonetheless, bearing witness to this process of opening up through dialogic exchanges gave many Trialogue participants hope for the future of mental health services.

“I was excited. My hope was restored in humanity. These people found the courage to come and to tell people what’s going on for them, and there’s more holistic understanding than I thought.” [IR.29]

#### Taking a risk: initial experiences of using Open Dialogue

The process of using Open Dialogue techniques to engage with others through Trialogue was initially quite challenging for many participants. Some participants indicated that they experienced initial difficulties in putting aside their assumptions about mental health. In particular, there were initial tensions that arose between service users and family members/carers on the one hand and service providers on the other, with the latter holding on to a medicalising view of service users.

“There are these three interest groups in the drama: there is family and friends and carers so to speak of, then there’s people who have experienced mental distress, and then there’s professionals who generally believe in this mental illness and they kind of generally are part of the system that medicalizes emotional and psychological difficulties. And, you know, everyone can be quite entrenched in their position like, and so the professionals [on the one hand] and the family and patients [on the other] will usually collude in terms of defining a situation.” [IR.40]

One participant also described the process of suspending assumptions as a risky venture as these assumptions often act as a form of protection.

“I think there is a risk as well. I think everybody is taking a chance, putting themselves out a limb in a way, to leave their hats at the door; maybe they are hard hats and they protect us, sometimes…” [FGR1.7]

Another participant expressed their initial nervousness about speaking at Trialogue meetings as they felt they had conflicting roles as both service provider and interested community member.

“I felt nervous about speaking at the Trialogue. I felt the ‘right’ thing to say as service provider might not be what came out if I was [playing the role of] the community member. I felt caught between the two roles a bit. I felt some embarrassment if someone asked me who I was and what I did as I felt I might be intruding or ‘spying’ even though I knew this was not the case.” [IR.42]

In spite of these initial tensions and difficulties, participants described how the option to remain anonymous in Trialogue Meetings afforded them with the opportunity to become more open and intimate with others.

“I also like the fact that you didn’t have to say who you are, where you are coming from, a little bit about yourself, you know? You can be as anonymous as you want [here] and that’s why I think people were more open.” [IR.7]

After the initial tensions subsided, one facilitator described how reluctance to talk gave way to a free-flowing dialogue, which was exciting but difficult to control.

“As it went on, it started to steam-roll and, when you have 40 people in a room – when it kicks off first, maybe [after] 20 minutes, nobody wants to say anything; and then all of a sudden, by the end of it, then you are trying to harness the whole thing. It was going out of control, you know? It’s quite exciting to be honest…” [FGR8]

#### “Something was beginning to happen”: the strengthening of bonds between Trialogue participants

A predominant concern raised by participants from the initial Trialogue Meetings was the lack of a sense of community for individuals with mental health problems in Ireland. Nonetheless, many participants felt that Trialogue Meetings went some way to bring people who deal with mental health systems together.

“Where you have people from different parts of the service, that engage in the service, coming together and talking about it; that’s a great idea.” [IR.15]

Some participants even noted that Trialogue Meetings were beginning to strengthen bonds between participants, some of whom had known each other previously.

“There are a few people I would have known, and friendships have really strengthened between us and we feel we can be more honest with each other because we actually spent time in the space and outside of the Trialogue there’s contact.” [FGR1.6]

This strengthening of bonds also contributed to a sense of shared ownership with the potential for generating its own energy for sustaining Trialogue.

“I think there is also a really interesting dynamic around ownership of the Trialogues because when you organize the Trialogues and you book the venue and, actually, you are just facilitating the Trialogue owning itself and it can choose. It can go a different way from your stated topic, for example, and you can bring it back but the Trialogue has its own energy that decides where it wants to go.” [FGR1.7]

Nonetheless, several participants identified that there was a need for all Trialogue Meeting participants to be more committed to the process and not to simply attend meetings without participating.

“I think if you are in that room, you have to be in that room for the very reasons that the Trialogue is about. You can’t just be there one week and be gone the next. It really has to have meaning because there is such a lot at stake, and for everybody who makes the effort to go there, that genuinely wants to be there.” [FGR1.11]

### Cycle 2

At this stage, the DCU team were beginning to hand over responsibility for running Trialogue meetings to local communities and a training day was set up to provide support for the communities in this transitional process. Even at this point, one of the Trialogue constituencies had branched off and formulated their own process and methodology, and, consequently, did not attend this training day.

#### “Telling it as it is”: The experience of becoming a Trialogue participant

Trialogue facilitators described engaging in Trialogue as an overwhelmingly positive experience for participants, with one facilitator identifying its potential to act as a platform for “coming out” about mental illness.

“It was the first space that was a kind of “coming out” for me, a very good space for me. I was surprised that I spoke that much about [my experience of the mental health services].” [FG2R.7]

Participating in Trialogue also led one facilitator to realize that finding solutions to problems through this process was less important than expressing oneself and sharing with others.

“What I’ve learnt from the Trialogue is that I used to feel that I had to have the answers and the solutions but the whole idea of the Trialogue is about sharing, not having the answers, about telling it as it is.” [FG2R.8]

A member of the project team also identified that the coffee breaks gave relatives an opportunity to speak outside the Trialogue discussions as they often find it difficult to give voice to their perspective.

“The relatives I have spoken to, I suppose, it might reflect what they find themselves between a rock and a hard place often in what their role is in the reality of the world […] It’s a complicated role and at the ends of the meetings, they are more or less saying more outside of the meeting rather than during the meetings.” [FG2R.2]

#### “Where do I come in?”: Solving teething problems with Open Dialogue

A number of facilitators identified initial teething problems which they encountered in their engagement in Trialogue Meetings. For instance, the initial excitement or energy from participating in Open Dialogue exchanges contributed to communication problems such as interrupting people.

“I get really excited about going to the Trialogue, and I get very excited within the Trialogue, and sometimes I might get over-excited and I crash into people and interrupt them and perhaps I shouldn’t.” [FG2R.8]

As a consequence, one facilitator identified the need for more experienced facilitators to moderate future Trialogue meetings in order to assist in open dialogic discussions.

“From the point of view of facilitating groups, I feel it’s important that the facilitator has some experience of facilitation, of watching the flow of the group. People [currently] aren’t offering very easily.” [FG2R.10]

Service providers who participated in Trialogue Meetings also experienced challenges in fighting against their inclinations to defend existing services and begun to listen more.

“I do think it’s probably a harder space for the service providers to speak than anyone else in the room because you do have a natural tendency to defend your service or defend what you do. But on the other hand, you have to try and let the real experience in the room, which is the people who have experienced the service, [you have to] try and take on board what they are saying and that’s the challenge for me personally.” [FG2R.5]

Nonetheless, the potential expressive space of Trialogue allowed participants to experience a liberating feeling of not knowing where the conversation might lead in Trialogue.

“I remember one night I had started off, I spoke and then I stopped and I just waited and I was amazed then: nobody came in, there was that space [to speak] and I just stopped and I experienced that [space]. That was probably one of the most striking things; that I could stop and the space would still be there for me and I could NOT know what I was going to say. I think if I could drop more into that space of not knowing, it would be more interesting and maybe more transformative as well.” [FG2R.7]

#### Strengthening bonds and taking responsibility: developing a community spirit with Trialogue

Focus group participants identified that the strengthening of bonds between Trialogue participants had led to the development of a strong community spirit. One individual described how this energised them, gave them greater compassion for others and enabled them to come out of their shell more.

“I’ve left the Trialogue feeling more energised and connected. I’d have feelings of compassion when I’d hear others’ stories, I’d feel touched and appreciative of them. […]Generally, at the end I would feel more connected and more expanded, that I had connected in some way with people, and I like that because I often take a withdrawn position.” [FG2R.7]

Another facilitator described how a growing sense of community spirit led to a transcendence of individual identities and kinship with others.

“There’s trust, intimacy and we’re building up relationships. Identity is only a level of human being, a superficial level of “Hi, you are this”. We’re actually community with awareness in the Trialogue group, so I find myself stepping beyond that and finding a mutuality, a universality.” [FG2R.8]

Nonetheless, a key issue that was raised by participants in the focus group was the need for communities to take greater ownership of the Trialogue Meetings. There was a sense that the DCU project team had been mainly responsible for getting Trialogue up-and-running and that it was now important for community members to take responsibility for Trialogue.

“The need to take ownership of the Trialogue group really came home to me so I’m going to take some personal responsibility now.” [FG2R.8]

### Cycle 3

The Cycle 3 data comprises material from the final Trialogue evaluation meetings, which involved the final hand-over of Trialogue from the DCU team to participating communities. In keeping with the spirit of anonymity of Trialogue, it was not possible to identify individuals associated with any given quotation. As such, each quotation in this section has only a Trialogue Evaluation Meeting [TEM] identifier associated with it.

#### Confidence through uncertainty: the experience of being a Trialogue participant

Participants in the final Trialogue Meetings described how they had begun to experience Trialogue as a non-threatening environment that allowed them to share information without fear or anxiety.

“Trialogue is a space where people using the services will divulge information without fear of judgement.” [TEM5]

“Knowledge is power. I was always quite fearful before, I am not quite so fearful as I was before. I am going to dump the word recovery and start using the word discovery.” [TEM1]

Furthermore, Trialogue participation built self-confidence among its members; notably, one participant suggested that this occurred as a result of the humility expressed by Trialogue participants.

“It’s how humble people can be that empowers others. Maybe that’s what you get at the Trialogue, to be humble and to listen to and to empower others.” [TEM5]

Another participant also identified that Trialogue participation invigorated them and provided them with a sense of freedom.

“I usually come out of Trialogue meetings feeling invigorated and connected. Also I often have a sense of freedom.” [TEM5]

#### Removing the mask: Becoming an open communicator

Trialogue participants identified how the process of using Open Dialogue and sharing different perspectives had enabled a discourse, which involved an open exchange of ideas, where no individuals attempted to exert power over others.

“I find that Trialogue is a unique experience of discourse within the “mental health” field. It is an open and equalitarian exchange of views and experiences of the participants. One is more normally exposed to a monologue which may (if the person hold’s power) be imposed without discourse.” [TEM1]

Adopting Open Dialogue methods also allowed participants to play with different roles and explore ideas in a context free from their normal assumptions.

“[Here I can play the role of] Democrat, libertarian, revolutionary; in an open and honest endeavour. This is a place where a mask can come off.” [TEM2]

One participant also expressed surprise at their abilities as a facilitator of Trialogue Meetings.

“I facilitated the last meeting, I never had done anything like that before, I surprised myself really. I got quite a lot out of facilitating, to allow others to express themselves.” [TEM5]

#### “Some kind of community”: establishing a community spirit with Trialogue

Participants from this cycle re-iterated the sense of community, inclusion and positive feelings they gained from Trialogue participation. One participant expressed how this community spirit meant that Trialogue had enabled them to approach issues relating to mental health in a spirit of creativity.

“I want to belong to a community [like Trialogue]. We co-create as vibrant beings, like notes on a piano.” [TEM2]

Another participant identified that this community spirit meant that Trialogue could act as a support structure for individuals after discharge from mental health services.

“One of the good things about it is it provides a space that helps people get back the power if they have been in hospital, once they get back into the community. Trialogue can help as a bridge back.” [TEM3]

Additionally, individuals who participated in the final Trialogue Meetings identified that the responsibility for sustaining Trialogue now lay in the hands of this new community but that this responsibility was an opportunity to develop its full potential.

“Handing it back to the community as you are doing now is very important.” [TEM2]

“Trialogue for me is an opportunity in itself to share in a neutral space opinions, experiences, beliefs about mental health.” [TEM2]

## Discussion

This is the first prospective study to purposefully describe the processes and challenges involved in participating in Trialogue Meetings from the perspective of participating individuals. The findings detail the evolving experiences of participants and track their engagement in Open Dialogue processes arising from their continuing participation in monthly Trialogue Meetings. In general, participants indicated across three Cycles of the research process that Trialogue Meetings provide a unique experience that was grounded in an empowering participatory approach, allowing them to develop key Open Dialogue skills and promoted a sense of community among them.

Regarding the Trialogue Meetings themselves, participants described them as comfortable spaces where they could express their feelings with less of a chance of being “railroaded” by others and, in some cases, led to a “coming out” about mental illness. These findings are striking in light of a recent systematic review which demonstrated that anxieties relating to disclosure are a pervasive barrier to help-seeking for mental health problems [26]. Since disclosure experiences can be both exhilarating and depressing for individuals with mental health problems [27], Trialogue Meetings may be a useful platform for individuals who are usually reticent to discuss their feelings about, and experiences of, mental health problems. Of particular note in this regard were the reports that relatives of service users found it easier to talk during the coffee breaks. Given that stigma has substantial negative psychosocial effects on the relatives of individuals with mental health problems [28], and family members often blame or self-stigmatize themselves in relation to a relative with mental health problems [29], these findings are heartening and suggest that Trialogue Meetings may be particularly helpful in integrating this under-represented group into mental health communities.

The current pattern of results also document participants’ development of key Open Dialogue skills across the study period. These findings resonate strongly with the skills and processes described by Bohm [30] as necessary for sustaining effective dialogue. For instance, in the first Trialogue Meetings, participants found it difficult to suspend their assumptions about mental illness, which led to some initial polarization between service providers’ medicalizing views of mental illness and a more humane approach from service users, family, friends and carers. Bohm [30] has proposed that such tensions are common initially for participants in a dialogue as individuals’ assumptions act as a “tacit” schema of reflexive thoughts through which they understand the world and autonomously produce knowledge, meaning that they often instinctively defend them when initially participating in a dialogue. Bohm [30] suggests that suspending these assumptions is important for effective dialogue as it allows ideas to reveal themselves without being coloured by particular worldviews. Nonetheless, while such polarization and defensiveness is inevitable when individuals initially participate in collective dialogue, this dissipates over time if participants are true to the spirit of the shared nature of the dialogic process. This process was clearly apparent in the current study among service providers, who initially felt compelled to defend existing mental health services. Over time, this natural defensiveness disappeared as these individuals began to listen more to others’ perspectives. These findings suggest that engaging in Trialogue Meetings may be particularly important for service providers in helping them to adopt a shared perspective with service users, community members and family members.

Participants also described the development of their dialogic skills across the study period, where their initial guarded series of exchanges gave way to open free-flowing conversations in which participants explored different roles and ideas and expressed confidence in not knowing where the conversation would lead. Bohm [30] has described how the initial “incoherence” of dialogue surrenders to coherent expressions of a collective and shared sense of meaning with practice. Importantly for participants, coherence and sensitivity regarding “when to come in” was aided by the rules of engagement for Trialogue such as the option for anonymity and the opportunity to speak without interruption. This coherence facilitated greater intimacy, openness and freedom in relation to where the conversation might lead. In this way, the Trialogue Meetings fulfilled the central purpose of dialogue from Bohm’s [30] perspective: to facilitate an individual to express themselves freely and truthfully. Indeed, this notion of self-expression relates to the etymological meaning of dialogue itself, which is derived from the Greek roots “dia” (through) and “logos” (the word); i.e. expressing something “through the word”.

Ultimately, this process of dialogic self-expression led to a number of positive outcomes for Trialogue participants such as lower anxiety and the dismantling of power dynamics between service users and providers. In the current study, participants indicated that these outcomes may have arisen due to the “transformative” power of self-expression in Trialogue Meetings. This explanation resonates with the French phenomenologist Maurice Merleau-Ponty’s [31] ideas that there is an existential element to the self-expression of speech in a dialogue, which corresponds to the artist’s, or musician’s, means of self-expression through a canvas or musical instrument. Most importantly for Merleau-Ponty, speech animates or vitalizes ideas and enables an individual’s thoughts to be brought to completion: “the thinking subject himself is in a kind of ignorance of his thoughts so long as he has not formulated them for himself” [31, p. 177]. According to Merleau-Ponty, the medium of speech brings ideas to life through the bodily expression of gesture, which allows one to explore others’ thoughts in an improvised and automatic (i.e. “tacit”) fashion which is fundamentally rooted in bodily expression. As such, the “vital” expression of speech brings with it an ability to think according to others and leads to the creation of a new “vital” and “shared” language. This enriches individuals’ own thoughts, facilitates a shared understanding or meaning between participants and enables a sense of universality or collectivity to emerge through dialogue: “As soon as man uses language to establish a living relation with himself or with his fellows, language is no longer an instrument, no longer a means; it is a manifestation, a revelation of intimate being and of the psychic link which unites us to the world and our fellow men.” [31, p. 196]. In sum, Merleau-Ponty’s ideas suggest that, where conditions for sustained self-expression are present (e.g. through Open Dialogue processes), participating in dialogue provides the individual with the opportunity to connect with a “vital” aspect of our being and share in “transformative” collective experiences with others. Such ideas may be particularly applicable to Trialogue Meetings, which have the potential for such a “vital” and “transformative” sense of self-expression according to participants from the current study.

In this way, Trialogue Meetings may facilitate better communication, and foster greater empathic experiences, between service users, service providers, community and friends/family members, allowing for greater integration between these groups in the delivery of mental health care. Indeed, Trialogue Meetings appear to offer an opportunity for individuals from formal mental health systems and local community settings to share experiences and improve communications in order to improve their care delivery towards service users; future research is needed to establish whether this could enable service providers to collaborate more effectively with community members and friends/family members involved in the care of service users. This approach may also be useful in other contexts, such as the delivery of care for individuals with chronic illnesses like heart disease or cancer, as a means to develop better communication and integrated care between individuals with chronic illnesses, health professionals involved in their care and friends/family members.

The current findings also trace the birth of a Trialogue community in Ireland across three cycles of Trialogue Meetings, from the initial strengthening of bonds between participants to a growing sense of kinship and the birth of a transcendent sense of collective identity. These developments mirror participatory definitions of community as social groups with a collective identity [4,5]. Participants also indicated that the development of a community spirit led to positive psychosocial outcomes such as increased social participation, elevated positive affect and formation of an arena for reciprocal psychosocial support. While the veracity of such findings requires quantitative validation in the context of Trialogue Meetings, they are in line with the consistent evidence of a positive relationship between community “social capital” and improved psychosocial outcomes in relation to mental health [e.g. 32,33,34]. They are also consistent with Baumann’s [35] conception of community as a safe place where individuals can rely on each other’s good will and can support each other in a spirit of co-operation. Indeed, participants identified with the support and good will associated with the development of a community spirit between service users, providers, family/carers and interested community members through Trialogue and explicitly described this as an empowering development. Lawson [36] has suggested that community empowerment through the development of a collective sense of identity has the potential to contribute to general wellbeing, greater equity and gaining resources and power to enable individual and collective goals to be actualized. Following such ideas, community-based participatory approaches such as Trialogue have the potential to promote recovery and empowerment among individuals from mental health contexts. This may have particular value for improving relations between relevant stakeholders who engage with mental health services, as there are significant power differentials that exist across such services [37,38].

Furthermore, the current pattern of findings suggests that the approach taken in the current study has the potential for sustainability in participating communities. Participants described how the process of handing over full responsibility and ownership for Trialogue to participating communities created an opportunity for community members to develop its full potential. It is worth noting that three of the seven participating communities have continued with Trialogue Meetings to the present day. Five further communities in Ireland have initiated Trialogue Meeting groups on a recurring or ad-hoc basis and the process and ethos of Trialogue Meetings has been incorporated into further community development training initiatives in all participating communities. These outcomes demonstrate the potential sustainability of Trialogue Meetings as a community-based participatory resource in healthcare contexts where Open Dialogue methods are desired and valued in order to strengthen relations and a sense of community between individuals in these contexts.

The current study is not without its limitations. In particular, the diverse data collection methods at each cycle of the research process may mean that the findings do not describe Trialogue Meeting processes in an exhaustive manner. Additionally, although participants were generally in agreement amount the processes of Trialogue, the current findings document their experiences of these processes rather than objectively documenting these processes themselves. Nonetheless, the credibility and value of the findings is evidenced by the variety of experiences described by participants. Furthermore, the flexible nature of the discussions in the interviews, focus groups and final Trialogue Meetings which constituted data from the three cycles of research allowed participants to raise issues of importance to them, while the independent analysis of the transcripts by a researcher who was not involved in the data collection phase ensured the analysis process was trustworthy.

## Conclusion

This is the first prospective study to purposefully describe the processes and challenges involved in participating in Trialogue Meetings. The findings demonstrate the usefulness of Trialogue Meetings as a community-based participatory approach which encourages disclosure and dialogue surrounding mental health, and has the potential to improve the integration of care between formal mental health systems and community care settings. Furthermore, the use of Open Dialogue skills and processes through Trialogue provides a platform for “vital” and “transformative” self-expression with the potential to result in lower anxiety, positive human contact and the dismantling of power dynamics for individuals from mental health communities. Trialogue Meetings also have the potential to be adapted for use in other healthcare contexts (e.g. individuals with chronic illnesses) in order to strengthen relations between individuals in these contexts and facilitate the growth of a spirit of community between them. In sum, where desirable conditions are present to allow for their effective development, Trialogue Meetings are a worthwhile and potentially sustainable community-based participatory approach.
